# Association of Obesity and Dietary Quality with Self-Reported Cardiovascular Disease Among Chinese Adults: A Cross-Sectional Study

**DOI:** 10.3390/nu18081241

**Published:** 2026-04-15

**Authors:** Panqi Wang, Gabriella Osgyáni-Balogh, Zsófia Verzár, Andrea Gubicskóné Kisbenedek

**Affiliations:** Department of Food and Nutrition Sciences, Institute of Nutritional Science and Dietetics, Faculty of Health Sciences, University of Pécs, 7621 Pécs, Hungary; gabibalogh01@gmail.com (G.O.-B.); verzar.zsofia@pte.hu (Z.V.); andrea@etk.pte.hu (A.G.K.)

**Keywords:** cardiovascular disease, dietary quality, health literacy, food frequency intake

## Abstract

**Background/Objectives:** Cardiovascular disease (CVD) is the leading cause of death in China. While obesity and dietary patterns are well-established factors, the independent association between overall dietary quality and CVD prevalence—specifically whether this link persists regardless of Body Mass Index (BMI)—requires further clarification. Furthermore, the behavioral and cognitive correlates that drive dietary quality, such as health literacy, remain insufficiently explored. This study evaluated the association of dietary quality with self-reported CVD among Chinese adults, independent of BMI, and identified the key behavioral and cognitive factors associated with dietary adherence in this population. **Methods:** This cross-sectional study surveyed 975 Chinese adults through anonymous questionnaires and collected self-reported data on CVD, BMI, dietary quality, and health literacy. One-way analysis of variance (ANOVA) and the chi-square test were used to compare the characteristics between groups, and multivariate Logistic regression was used to analyze the association between dietary quality and the odds of CVD, sequentially adjusting for variables such as BMI, physical activity. **Results:** Higher dietary quality was independently associated with lower odds of CVD (Model 3: OR = 0.879, 95% CI: 0.845–0.915, *p* < 0.001). Notably, this inverse association remained significant after adjusting for BMI, which itself showed no significant association with CVD prevalence in the multivariable model. Regarding population profiling, poor dietary quality was significantly related to regular smoking (*p* < 0.05), whereas age, gender, residence, employment status, and BMI showed no significant associations with dietary quality categories. Furthermore, health literacy (*p* < 0.05) and physical activity (*p* < 0.05) showed positive associations with superior dietary quality. **Conclusions:** Dietary quality is a significant independent factor inversely associated with CVD prevalence, regardless of obesity status. Suboptimal dietary habits cluster among smokers and individuals with lower health literacy and physical activity levels, showing a stronger association with cognitive and behavioral factors than with demographic or occupational characteristics. Interventions should prioritize enhancing health literacy and addressing the clustering of unhealthy behaviors to effectively address the cardiovascular burden in the Chinese population.

## 1. Introduction

Cardiovascular diseases (CVDs) are the most serious challenge facing global public health in the 21st century. Despite significant progress in drug and surgical treatments, CVDs remain the leading cause of premature death and the continuous rise in medical expenses worldwide. In China, the epidemiological transformation from infectious diseases to chronic non-communicable diseases has been rapid and substantial. According to the 2024 annual report on cardiovascular health and diseases in China: Data and trend, the number of cases, incidence rate and standardized incidence rate of CVD in the entire Chinese population rose from 5.3007 million, 447.81 per 100,000 and 646.20 per 100,000 in 1990 to 12.3411 million, 867.65 per 100,000 and 652.21 per 100,000 in 2019, respectively. The standardized incidence rate of CVD among Chinese residents from 1990 to 2019 generally showed an upward trend. The predicted incidence rate, predicted mortality rate and predicted disability-adjusted life year (DALY) rate of CVD among Chinese residents from 2020 to 2030 all continued to rise, especially in regions with an aging population and insufficient medical services [1].

In recent years, the prevalence of overweight and obesity among Chinese adults has continued to rise. From 2013 to 2018, the standardized prevalence of obesity and overweight among Chinese adults increased from 19.3% to 25.6%, and the prevalence of obesity rose from 3.1% to 8.1%. A cross-sectional study of 15.7 million adults in 2019 showed that the prevalence rates of overweight and obesity were 34.8% and 14.1%, respectively. In addition, complications such as fatty liver, prediabetes, dyslipidemia and hypertension are more common among overweight and obese people, and the likelihood of these complications also increases significantly with the increase in BMI. These trends highlight the challenges obesity poses to public health in China and suggest the need to formulate targeted intervention measures [2,3]. However, an increasing amount of evidence indicates that the relationship between weight and cardiovascular health is not always linear. Therefore, increasing attention has been directed toward “metabolic health” rather than just BMI.

In tandem with the obesity epidemic, China is undergoing a profound nutritional transformation. The traditional diet pattern, which mainly consists of high-fiber plant-based foods, is rapidly being replaced by the “Western” diet pattern, characterized by high sodium, refined carbohydrates and saturated fats. A study published in JMIR Public Health and Surveillance in 2025 systematically evaluated the disease burden caused by dietary factors among Chinese adults aged 25 and above. Research has found that in 2021, diet-related factors were associated with approximately 1.7 million deaths and 38.39 million disability-adjusted life years (DALYs), with CVD being the main contributor. High sodium intake and low intake of fruits and whole grains are the most significant dietary factors. In addition, the increased consumption of red meat and sugary drinks has led to a significant rise in the burden of related diseases [4]. These trends emphasize the urgency of formulating comprehensive and adaptable nutrition policies to address the changing burden of diet-related diseases in China.

Emerging evidence emphasizes that an overall dietary pattern can more comprehensively reflect health outcomes than a single nutrient. Specifically, a recent large-scale prospective study of Chinese adults has shown that a higher Global Dietary Quality Score (GDQS, an indicator emphasizing nutrient-intensive plant-based foods) is significantly and inversely associated with a lower odds of non-fatal CVD and all-cause mortality [5]. These findings suggest that optimizing the quality of dietary patterns may be a key strategy for cardiovascular protection in the Chinese population.

In this connection, the role of health literacy and socio-economic constraints is of vital importance, yet it is often overlooked. In modern Chinese society, full-time employees are increasingly confronted with the predicament of “time poverty”, which is gradually being regarded as an important social determinant affecting health. Work pressure leaves them with almost no time to prepare healthy food or engage in physical exercise. Research shows that among urban laborers, Women, low-income workers, married people and those who need to take on care responsibilities are more likely to fall into the predicament of insufficient time, and lower minimum wage standards and higher overtime rates will further exacerbate this problem. The evidence from a systematic review further indicates that structural factors such as long working hours, unpaid labor, commuting burdens, and work disruptions are associated with less time spent on preparing a healthy diet, engaging in physical exercise, and restoring their physical and mental health, and are linked to a higher prevalence of adverse health outcomes [6,7]. Furthermore, having received formal education does not necessarily mean having sufficient health literacy—that is, the ability to obtain, understand and apply health information to make informed dietary choices. Previous studies have mainly focused on a single associated factor, and few have combined cognitive drivers such as health literacy with behavioral outcomes such as dietary quality and clinical outcomes such as CVD. Recent studies have shown that people with higher health literacy exhibit stronger dietary self-efficacy and are more inclined to use nutrition labels, suggesting a potential pathway that links cognitive resources with behavioral patterns and cardiovascular health status [8].

Therefore, it is urgently necessary to study the aggregation of these factors among the contemporary Chinese population. This cross-sectional study aims to fill these research gaps. Specifically, our objectives are as follows: (1) to explore the independent and combined associations between obesity and dietary quality and self-reported CVD; (2) investigate whether a high-quality diet is associated with lower cardiovascular prevalence typically associated with a high BMI; (3) identify the sociodemographic and cognitive determinants that lead to poor dietary quality, such as employment status and health literacy. By clarifying these complex correlations, this study provides a scientific basis for formulating more detailed public health policies targeting the prevention of CVD in China.

## 2. Materials and Methods

### 2.1. Research Design and Data

This study employed a cross-sectional design, with data sourced from a self-administered questionnaire survey conducted among adults in mainland China between September and November 2024. The survey aimed to explore the relationship between dietary habits, lifestyle factors, and health status with CVD prevalence.

Participants were recruited using convenience sampling through an online survey platform. Inclusion criteria were adults aged ≥18 years who were able to complete the questionnaire independently and signed an informed consent form before participation. Individuals who did not provide complete responses to key variables (including age, body mass index (BMI), or CVD status) were excluded from the final analysis.

This study used a self-designed Chinese Dietary Quality Survey. The questionnaire contained 61 items, divided into six parts: basic demographic information (12 items, such as gender, age, height and weight), health literacy issues (13 items, such as whether one can understand medical advice and read drug instructions), health issues (4 items, based on whether one regularly participates in disease screening and whether one has circulatory system diseases), physical activity (7 items, such as the duration of different types of exercise per week), dietary habits (20 items, such as food frequency questionnaire), and food choice attitudes (4 items, such as factors influencing food choices). Depending on the content of the questions, the questionnaire used either categorized options or frequency options for responses.

This study also recognizes the limitations of convenience sampling in terms of representativeness and extrapolation. Therefore, the findings should be interpreted with caution when generalizing the research.

All participants signed the informed consent form before participating. This survey was conducted anonymously and does not collect any information that directly identifies individuals.

### 2.2. Data Collection Methods

#### 2.2.1. Assessment of Cardiovascular Disease

The primary outcome of this study was self-reported CVD, determined through a standardized survey. Participants were asked to indicate whether they had ever been diagnosed with any cardiovascular conditions. For the purposes of this analysis, CVD was defined as including hypertension, ischemic heart disease, coronary heart disease, stroke, and myocardial infarction, among others. Participants who reported a physician-confirmed diagnosis of at least one of these conditions were categorized as having CVD.

#### 2.2.2. Anthropometric Measurements

Height and weight data were self-reported by the participants through anonymous questionnaires. To enhance the accuracy of the data, participants were required to fill in the values of their height and weight obtained from their most recent medical measurements. The body mass index (BMI) was calculated by researchers based on a standard formula, which is weight (kg) divided by the square of height (m) (kg/m^2^). In addition, to minimize potential confounding, pregnant women and participants with atypical body compositions (e.g., professional athletes) were excluded from the analysis. To enhance the clinical relevance of the research results, the participants were grouped according to the BMI classification criteria for Chinese adults: Underweight (BMI ≤ 18.5 kg/m^2^), normal weight (18.5 ≤ BMI < 24.0 kg/m^2^), overweight (24.0 ≤ BMI < 28.0 kg/m^2^), and obesity (BMI ≥ 28.0 kg/m^2^) [9].

#### 2.2.3. Dietary Assessment and Scoring

Dietary intake was assessed using a 33-item food frequency questionnaire (FFQ), which was adapted from the validated 64-item instrument used in the 2015 China Adult Chronic Disease and Nutrition Surveillance (CACDNS 2015) [10]. The 33 items were specifically selected to reflect the core dietary patterns and nutritional landscape of the Chinese population. While the item list was streamlined to enhance participant compliance in an online setting and minimize respondent fatigue by excluding less relevant or redundant food categories, the scoring framework remained strictly aligned with the evidence-based principles of the “Chinese Dietary Guidelines (2022)” [11]. To ensure the reliability of this adapted version within our study sample, internal consistency was evaluated using Cronbach’s alpha coefficient. The scoring followed a standardized protocol: higher scores were assigned to nutrient-dense foods recommended for regular consumption, while lower scores were given to those recommended for limited intake, thereby ensuring that the final score objectively represents adherence to national nutritional standards. Although the adapted FFQ was not independently re-validated in this study, it is based on a nationally validated framework, which ensures the structural validity of the food categories and assessment items. In the current sample, the 33-item FFQ demonstrated excellent internal reliability, with a Cronbach’s alpha of 0.980.

The 33 foods were categorized into three groups: Healthy foods (10 types), including milk, dairy products, fresh vegetables, fresh fruits, legumes, legume products, fish, whole-grain cereals, dried fruit and poultry.; Neutral foods (8 types), including lean pork, beef, rice, pasta products, bread, potatoes, animal liver, and cheese.; and Unhealthy foods (15 types), foods that should be limited in intake, including high-salt, high-oil, high-sugar, and processed foods, including fatty pork, other animal organs, sausages, smoked meat products, cream, butter, fried pasta products, fried potatoes, pickled vegetables, ice cream, fast food, convenience foods, canned fruit, canned fish and semi-finished products.

A six-point frequency scale was used (multiple times daily, once daily, several times weekly, several times monthly, occasionally, never), and different scoring rules were applied for each category. Healthy foods were scored positively (2, 1, 0), neutral foods were scored moderately (1 or 0), and unhealthy foods were scored in reverse (2, 1, 0). The total dietary quality score ranged from 0 to 66, with higher scores indicating better adherence to dietary guidelines.

Participants were divided into tertiles based on the distribution of dietary quality scores (tertile categorization based on the distribution of dietary quality scores): low (T1), moderate (T2), and high (T3), ensuring balanced group sizes. One-way ANOVA was used for continuous variables, and chi-square tests were used for categorical variables.

### 2.3. Assessment of Covariates

Sociodemographic factors and lifestyle factors were collected as covariates. These factors include age, gender, place of residence (urban/rural), educational attainment, marital status (married/single) and employment status (divided into full-time and other). Lifestyle variables include smoking status (never smoked, quit smoking or currently smoking), alcohol consumption and physical activity level (frequency and duration).

Physical activity data assessment: Physical activity levels were quantified using the validated International Physical Activity Questionnaire (IPAQ) short-question core item assessment method [12]. The questionnaire collected data on the number of days participants engaged in vigorous exercise, moderate exercise, and walking in the past week, and the duration of each session (options: <0.5 h, 0.5–1 h, 1–1.5 h, 1.5–2 h, >2 h). For comprehensive analysis, the duration categorical variable was first converted to median values (15, 45, 75, 105, and 135 min, respectively), and the total weekly minutes for each intensity of activity were calculated. Then, based on common standards for energy expenditure in physical activity, corresponding metabolic equivalents (METs) were assigned to each intensity: vigorous exercise 8.0 METs, moderate exercise 4.0 METs, and walking 3.3 METs. The weekly MET-minutes for each intensity of activity were calculated using the formula (weekly exercise minutes × MET value). An individual’s overall physical activity level was expressed as the total weekly MET-minutes, which is the sum of the MET-minutes for all intensities. In the subsequent multivariate analysis, this overall physical activity level was treated as a continuous variable to maintain maximum statistical power.

Health Literacy Assessment: Health literacy was evaluated using of the European Health Literacy Questionnaire (HLS-EU-Q). The conceptual framework of this questionnaire is based on the multi-dimensional model proposed by Sørensen et al. [13]. To ensure data integrity and minimize neutral response bias, a four-point mandatory selection Likert scale was adopted: “very difficult” (1), “relatively difficult” (2), “relatively easy” (3), “very easy” (4) and “I don’t know/not applicable” (5). The option “don’t know” was deliberately omitted to encourage participants to assess their ability to search for, understand, judge and apply health information. The total score of health literacy ranges from 13 to 52 points. The higher the score, the higher the functional health literacy.

Statistical Analysis Strategy: All statistical analyses were conducted on the raw data derived from the convenience sample. To maintain the integrity of the associations observed within this specific study population and to avoid potential data distortion—given the immense geographical and socioeconomic diversity in dietary habits across China—no post-stratification weighting or adjustment to national demographics was performed. The findings are intended to reflect dietary and health patterns specific to the current sample.

### 2.4. Statistical Analysis

Continuous variables were expressed as mean ± standard deviation (SD), and one-way analysis of variance (ANOVA) was used to compare the differences among different tertile groups of dietary quality. Categorical variables were expressed as frequency and percentage (n, %), and analyzed using the chi-square (χ^2^) test. To explore the association between dietary quality and the CVD status, a multivariate logistic regression model was used to calculate the odds ratio (OR) and 95% confidence interval (CI). Three models were constructed: Model 1: Unadjusted model; Model 2: Adjust for sociodemographic factors (age, gender, place of residence and educational attainment); Model 3: On this basis, further adjust the body mass index (BMI), smoking, drinking, and physical activity. All statistical analyses were conducted using SPSS version 22.0 software. A bilateral *p* value < 0.05 was considered statistically significant.

## 3. Results

### 3.1. Descriptive Characteristics of the Study Population

A total of 1000 questionnaires were collected in this study. After eliminating incomplete or invalid questionnaires, a total of 975 valid questionnaires were finally included, with a valid response rate of 97.5%. The baseline characteristics of the research subjects are as follows: There were 515 males and 460 females in the sample, with an average age of 40 ± 12.605 years. Among them, 22.4% (n = 218) of the research subjects suffered from CVD, and 77.6% (n = 757) of the research subjects did not have CVD. The dietary assessment tool showed high reliability, with a Cronbach’s alpha coefficient of 0.980 for the 33-item FFQ.

Table 1 summarizes the socio-demographic and health-related characteristics of 975 participants, with a focus on their prevalence of CVD. Overall, in terms of categorical variables, gender had no significant association with CVD. The BMI was not significantly associated with CVD. Smoking behavior was significantly related to CVD. Although there was no statistically significant association between drinking behavior and CVD, marital status was highly correlated with CVD. Educational background was significantly correlated with CVD. Place of residence was significantly related to CVD. Occupation status was not significantly related to CVD. Regarding continuous variables, in terms of age, the mean age of CVD patients was significantly higher than that of the non-CVD group. Secondly, in terms of physical activity (expressed as total weekly MET-minutes, MET), the mean level of CVD patients was significantly lower than that of the non-CVD group. Regarding dietary quality scores, the mean scores of CVD patients were also significantly lower than those of the non-CVD group. Finally, regarding health literacy score, the mean scores of CVD patients were significantly lower than those of the non-CVD group.

### 3.2. Independent Association Between Dietary Quality and CVD

Multivariable logistic regression analysis was used to evaluate the independent association between dietary quality score and CVD (Table 2). In the crude model (Model 1) that only included the dietary quality score, the dietary quality score was significantly inversely associated with the odds of CVD. After adjusting for sociodemographic factors and BMI (Model 2), this association remained significant. In the fully adjusted model (Model 3) that further included lifestyle and health-related variables, the inverse association between dietary quality score and CVD odds still existed.

### 3.3. Determinants of Dietary Quality and Population Profiling

To characterize participants based on their dietary patterns, demographic, socioeconomic, and lifestyle factors were compared across dietary quality tertiles (Table 3). The analysis indicated that dietary quality in the studied population was primarily differentiated by behavioral and cognitive factors rather than biological or fixed demographic characteristics. A significant association was observed between dietary quality and smoking status. Regarding tobacco use, regular smokers were more prevalent in the lowest tertile than in the highest tertile, whereas never-smokers were most likely to belong to the highest dietary quality group. Furthermore, the results of the one-way ANOVA demonstrated highly significant differences in physical activity levels and health literacy scores across the three groups. Post hoc comparisons indicated that individuals in the moderate and high dietary quality groups exhibited significantly higher health literacy scores than those in the lowest group. Similarly, physical activity levels (MET) were significantly higher in the moderate dietary group compared to the lowest group. In contrast, no statistically significant differences were observed across the dietary quality tertiles regarding age, gender, marital status, education level, employment status, residence, alcohol consumption, or BMI. These findings suggest that suboptimal dietary quality is a widespread issue across different age groups, genders, and weight categories in this population, remaining independent of their residential environment, educational background, or employment status. Detailed data can be found in Table 3.

## 4. Discussion

### 4.1. Principal Findings

The core finding of this study is that a higher dietary quality score is significantly related to lower odds of CVD. Our observed association (OR = 0.879, 95% CI: [0.845–0.915]) is consistent with the cross-sectional findings of Gao et al. [14], who utilized data from the China Health and Nutrition Survey (CHNS). Their study similarly demonstrated that higher diet quality was significantly associated with lower odds of stroke among Chinese adults (e.g., OR = 0.42 for the highest diet quality category in women). While our study observed a relatively more modest effect, both sets of results consistently suggest that superior dietary quality is associated with lower cardiovascular prevalence in the Chinese population. The key significance lies in the fact that even after fully adjusting for traditional factors such as BMI, smoking, physical activity, this association remains robust. This suggests that dietary quality is an independent correlate of CVD status, regardless of BMI status. Previous studies have pointed out that a suboptimal dietary structure is a primary controllable factor for the CVD burden in the Chinese population [4]. This study, based on data from 975 adults, further supports the consistency of this inverse association among individuals with different weight bases.

### 4.2. Biological Mechanisms and the Interaction with Obesity

The observed inverse association between dietary quality and CVD prevalence is consistent with established biological mechanisms. A high-quality diet provides essential dietary fiber, antioxidants, and unsaturated fatty acids, which are linked to attenuated systemic inflammation and oxidative stress—key pathways in the pathogenesis of atherosclerosis. While obesity remains a well-established independent correlate of heart disease in the Chinese population [15], this study found no significant association between BMI and CVD prevalence. This might be attributed to potential self-reporting bias or the relatively young age of the sample, where BMI variance may not yet be fully reflected in clinical status. Crucially, our multivariable analysis demonstrated that the cardiovascular health correlates of a healthy diet persist independently of weight status. This suggests that dietary patterns may serve as a metabolic ‘buffer’ against the pro-inflammatory state associated with excess adiposity. For instance, high-quality dietary intake is associated with better insulin sensitivity and vascular endothelial function even in overweight or obese individuals, potentially offsetting some of the adverse cardiovascular correlates associated with adipokines.

### 4.3. Determinants of Dietary Quality

Lifestyle and cognitive correlates: A unique finding of this study is the identification of specific behavioral and cognitive characteristics associated with dietary quality. Unlike traditional demographic factors such as age, place of residence, or employment status (which showed no significant associations in this sample), smoking status and physical activity emerged as key correlates [16]. This suggests that dietary habits are more closely linked to modifiable lifestyle behaviors rather than fixed socioeconomic backgrounds. Furthermore, the significant clustering of smoking and poor dietary quality supports the “clustering of unhealthy behaviors” hypothesis, while higher levels of physical activity were found to coexist with better dietary adherence. More importantly, there is a significant positive association between health literacy and dietary quality, highlighting the cognitive dimension of nutritional behavior. Although educational level was not a significant factor in our analysis, health literacy—a measure of the ability to process and apply health information—was a prominent correlate associated with dietary quality [17]. This suggests that merely providing “knowledge” may be insufficient; instead, improving functional health literacy may be pivotal in bridging the gap between nutritional awareness and actual dietary practice.

### 4.4. Strengths and Limitations

This study has several advantages, including a comprehensive assessment of dietary quality and health literacy within a large sample of Chinese adults. However, some limitations must be acknowledged. First, as a cross-sectional study, we cannot infer causality or rule out the possibility of reverse causation. For instance, individuals diagnosed with CVD may have proactively improved their dietary habits post-diagnosis. Such behavioral changes could confound the observed association between dietary quality scores and CVD prevalence. Additionally, since the specific duration since CVD diagnosis was not recorded in our survey, we were unable to perform sensitivity analyses to exclude recent diagnoses, which is a common limitation in cross-sectional nutritional research. Second, while the 33-item FFQ used in this study was adapted from the validated framework of the 2015 China Adult Chronic Disease and Nutrition Surveillance (CACDNS 2015) [10], the shortened version itself has not been independently re-validated as a standalone instrument in this specific population. However, the internal consistency (Cronbach’s alpha) of the 33-item FFQ was calculated as 0.980 in our sample, indicating excellent reliability. The use of items from an established national surveillance tool enhances the structural validity of our dietary assessment. Furthermore, as the FFQ focused solely on consumption frequency to ensure participant compliance, the inherent recall bias remains, although this is a common challenge in large-scale nutritional epidemiology. Third, participants were recruited via an online platform using convenience sampling, which inevitably introduced selection bias. Online survey participants often possess higher digital literacy and tend to be younger and more educated than the general Chinese population. Consequently, our sample may not fully represent groups with limited digital access, such as the elderly or residents in remote rural areas. Furthermore, we did not apply weighting or post-stratification adjustments to match national demographics. Given the vast geographical and socioeconomic diversity in dietary habits across China, weighting by national averages might have introduced further data distortion; thus, our findings should be interpreted specifically within the context of this convenience sample. Future research should employ multi-stage stratified random sampling and longitudinal designs to enhance the generalizability and causal clarity of these findings across diverse demographic segments in China. Fourth, since the data were collected through a self-administered online questionnaire, the findings are subject to social desirability bias. Participants may have intentionally or unintentionally “improved” their reported dietary frequencies based on their nutritional knowledge. Consequently, without external professional supervision to validate the reported frequencies, these self-reported data should be interpreted as informational and indicative of dietary trends within this population, rather than absolute clinical measurements. Fifth, BMI was modeled using traditional linear approaches in the fully adjusted analysis. However, we acknowledge that potential non-linear relationships (e.g., J-shaped associations) and age-dependent effects were not examined using more advanced methods such as restricted cubic spline modeling or age-stratified analyses. This may limit the comprehensiveness and interpretability of the findings regarding the relationship between body mass and CVD. Finally, since the specific duration since CVD diagnosis was not recorded in our survey, we were unable to perform sensitivity analyses to exclude recent diagnoses or to stratify the results by the time elapsed since diagnosis. Consequently, the possibility of reverse causation—where participants may have adopted healthier diets following their diagnosis—cannot be fully ruled out. If such dietary improvements occurred, they would likely bias the observed associations toward the null, potentially leading to an underestimation of the true relationship between diet quality and CVD. We acknowledge this as a limitation and suggest that future studies should incorporate diagnosis timing to better elucidate these temporal relationships.

## 5. Conclusions

In conclusion, this study indicates that dietary quality is a significant independent factor associated with cardiovascular disease (CVD) prevalence in Chinese adults, and this association is independent of an individual’s obesity (BMI) status. Research found that poor dietary behaviors show significant clustering among specific groups, primarily smokers, individuals with lower physical activity, and those with limited health literacy. This suggests that dietary habits are closely intertwined with both lifestyle patterns and cognitive abilities, rather than being strictly tied to fixed demographic or occupational characteristics. Therefore, future public health strategies should move beyond general dietary advice to focus on precise, targeted interventions. By enhancing the health literacy of the population and addressing the clustering of unhealthy behaviors, it may be possible to more effectively address the overall burden of cardiovascular diseases in China. Given the cross-sectional nature of this study, prospective cohort studies are warranted to further explore the potential causal pathways underlying these findings.

## Figures and Tables

**Table 1:** Baseline characteristics of the study participants stratified by CVD status.

Variables	Category	CVD		Total n (%)	t/χ^2^	*p* Value
		Non-CVD (n = 757)	CVD (n = 218)			
Age (years, mean ± SD)	-	37.90 ± 12.496	47.28 ± 10.027	-	−11.476	**<** **0** **.001**
Gender, n (%)	Male	403 (53.2%)	112 (51.4%)	515 (52.8%)	0.235	0.628
	Female	354 (46.8%)	106 (48.6%)	460 (47.2)		
BMI	Underweight	100 (13.2%)	23 (10.6%)	123 (12.6%)	2.054	0.561
	Normal weight	373 (49.3%)	104 (47.7%)	477 (48.9%)		
	Overweight	213 (28.1%)	66 (30.3%)	279 (28.6%)		
	Obese	71 (9.4%)	25 (11.5%)	96 (9.8%)		
Education, n (%)	Primary	193 (25.5%)	38 (17.4%)	231 (23.7%)	8.436	**0.015**
	Secondary	240 (31.7%)	88 (40.4%)	328 (33.6%)		
	College	324 (42.8%)	92 (42.2%)	416 (42.7%)		
Marital status, n (%)	Married	491 (64.9%)	173 (79.4%)	664 (68.1%)	16.374	**<** **0** **.001**
	Single	266 (35.1%)	45 (20.6%)	311 (31.9%)		
Residence, n (%)	Urban	592 (78.2%)	153 (70.2%)	745 (76.4%)	6.040	**0.018**
	Rural	165 (21.8%)	65 (29.8%)	230 (23.6%)		
Occupation, n (%)	Full-time	395 (52.2%)	124 (56.9%)	519 (53.2%)	1.604	0.448
	Part-time	132 (17.4%)	36 (16.5%)	168 (17.2%)		
	Not employed	230 (30.4%)	58 (26.6%)	288 (29.5%)		
Smoking Behavior, n (%)	Regular smoker	197 (26.0%)	75 (34.4%)	372 (27.9%)	8.401	0.015
	Occasional smoker	246 (32.5%)	52 (23.9%)	298 (30.6%)		
	Never smoker	314 (41.5%)	91 (41.7%)	405 (41.5%)		
Drinking Behavior, n (%)	Regular drinker	130 (17.2%)	52 (23.9%)	182 (18.7%)	5.287	0.071
	Occasional drinker	238 (31.4%)	59 (27.1%)	297 (30.5%)		
	Never drinker	389 (51.4%)	107 (49.1%)	496 (50.9%)		
Total MET (mean ± SD)	-	4134.04 ± 3074.768	3096.31 ± 2551.464	-	5.043	**<** **0** **.001**
Dietary Quality Score (mean ± SD)	-	33.72 ± 4.874	31.47 ± 4.238	-	6.688	**<** **0** **.001**
Health Literacy Score (mean ± SD)	-	36.15 ± 9.703	29.18 ± 9.283	-	9.431	**<** **0** **.001**

Continuous variables were expressed as mean ± standard deviation and compared using the independent sample *t*-test. Categorical variables were expressed in terms of frequency (percentage) and compared using the Pearson chi-square test. Bold values indicate statistical significance (*p* < 0.05) based on the chi-square test.

**Table 2:** Association between dietary quality score and the odds of CVD (N = 975).

Models	OR	95% CI	*p* Value
Model 1 (Unadjusted)	0.895	0.863–0.928	**<0.001**
Model 2 (Adjusted^1^)	0.880	0.846–0.916	**<0.001**
Model 3 (Adjusted^2^)	0.879	0.845–0.915	**<0.001**

Adjusted^1^ for age, gender, marital status, education level, occupation, residence, and BMI (continuous). Adjusted^2^ for age, gender, education level, marital status, occupation, residence, BMI, smoking status, alcohol consumption and total physical activity (MET). Bold values indicate statistical significance (*p* < 0.05) based on the chi-square test.

**Table 3:** Distribution of demographic, socioeconomic, and lifestyle factors across tertiles of dietary quality scores (N = 975).

Variables	Category	T1 (Low)	T2 (Middle)	T3 (High)	*p* Value
Age (years)	-	39.62 ± 12.722	40.25 ± 12.566	40.16 ± 12.544	0.781
Gender, n (%)	Male	185 (53.3%)	172 (55.1%)	158 (50.0%)	0.425
	Female	162 (46.7%)	140 (44.9%)	158 (50.0%)	
Residence, n (%)	Urban	261 (75.2%)	231 (74.0%)	253 (80.1%)	0.166
	Rural	86 (24.8%)	81 (26.0%)	63 (19.9%)	
Education, n (%)	Primary	90 (25.9%)	77 (24.7%)	64 (20.3%)	0.298
	Secondary	119 (34.3%)	105 (34.0%)	103 (32.6%)	
	College	138 (39.8%)	129 (41.3%)	149 (47.2%)	
Occupation, n (%)	Full-time	182 (52.4%)	169 (54.2%)	168 (53.2%)	0.961
	Part-time	64 (18.4%)	51 (16.3%)	53 (16.8%)	
	Not employed	101 (29.1%)	92 (29.5%)	95 (30.1%)	
Marital status, n (%)	Married	241 (69.5%)	200 (64.1%)	223 (70.6%)	0.176
	Single	106 (30.5%)	112 (35.9%)	93 (29.4%)	
Smoking Behavior, n (%)	Regular smoker	112 (32.3%)	84 (26.9%)	76 (24.1%)	**0.01**
	Occasional smoker	113 (32.6%)	99 (31.7%)	86 (27.2%)	
	Never smoker	122 (35.2%)	129 (41.3%)	154 (48.7%)	
Drinking Behavior, n (%)	Regular drinker	71 (20.5%)	60 (19.2%)	51 (16.1%)	0.468
	Occasional drinker	111 (32.0%)	92 (29.5%)	94 (29.7%)	
	Never drinker	165 (47.6%)	160 (51.3%)	171 (54.1%)	
Total MET (mean ± SD)	-	3895.97 ± 2919.016	4346.15 ± 3360.025	3470.14 ± 2620.158	**0.001**
BMI	-	23.358 ± 6.9410	22.875 ± 4.6288	23.361 ± 3.9180	0.425
Health Literacy Score (mean ± SD)	-	33.14 ± 10.846	35.73 ± 10.703	35.07 ± 8.079	**0.00** **2**

Bold values indicate statistical significance (*p* < 0.05) based on the chi-square test.

## Data Availability

The data presented in this study are available from the corresponding author upon reasonable request. The data are not publicly available due to privacy and ethical restrictions.

## Abbreviations

The following abbreviations are used in this manuscript:

CVD	Cardiovascular disease
FFQ	Food Frequency Questionnaire
MET	Metabolic Equivalent of Task
BMI	Body Mass Index
